# Adolescent fluoxetine exposure increases ERK-related signaling within the prefrontal cortex of adult male Sprague-Dawley rats

**DOI:** 10.1093/oons/kvac015

**Published:** 2022-10-20

**Authors:** Anapaula Themann, Minerva Rodriguez, Israel Garcia-Carachure, Omar Lira, Sergio D Iñiguez

**Affiliations:** Department of Psychology, The University of Texas at El Paso, 500 University Ave., El Paso, TX 79968, USA; Department of Psychology, The University of Texas at El Paso, 500 University Ave., El Paso, TX 79968, USA; Department of Psychology, The University of Texas at El Paso, 500 University Ave., El Paso, TX 79968, USA; Department of Psychology, The University of Texas at El Paso, 500 University Ave., El Paso, TX 79968, USA; Department of Psychology, The University of Texas at El Paso, 500 University Ave., El Paso, TX 79968, USA

**Keywords:** RSK, mTOR, MAPK, long-term, ERK, antidepressant

## Abstract

There has been a disproportionate increase in fluoxetine (FLX) prescription rates within the juvenile population. Thus, we evaluated how adolescent FLX exposure alters expression/phosphorylation of proteins from the extracellular signal-regulated kinase (ERK)-1/2 cascade within the adult prefrontal cortex (PFC). Male Sprague-Dawley rats were exposed to FLX (20 mg/kg) for 15 consecutive days [postnatal day (PD) 35–49]. At PD70 (adulthood), we examined protein markers for ERK1/2, ribosomal S6 kinase (RSK) and mammalian target of rapamycin (mTOR). FLX-pretreatment decreased body weight, while increasing PFC phosphorylation of ERK1/2 and RSK, as well as total mTOR protein expression in adulthood. We provide first-line evidence that juvenile FLX pretreatment induces long-term decreases in body weight gain, along with neurobiological changes in the adult PFC—highlighting that early life antidepressant exposure increases ERK-related signaling markers in later life.

## INTRODUCTION

Adolescence, the transitional stage between childhood and adulthood, is the developmental period wherein the first incidence of depression is most often reported. Given its chronic and recurring nature, the prevalence of depression remains a major public health issue across the globe; particularly post-COVID-19 pandemic [1]. One of the most common pharmacological treatments for the management of depression, particularly in the juvenile population, is administration of the selective serotonin reuptake inhibitor (SSRI) fluoxetine (FLX). Since FLX is one of the few SSRI’s approved by the United States Food and Drug Administration for the treatment of pediatric depression, it is not surprising that the prescription rates of this antidepressant medication are high [2]. Although FLX has been regarded efficacious for the treatment of mood-related illnesses in children and adolescents, its long-term molecular effects have not been thoroughly assessed [3, 4]—predominantly within young populations [5, 6]. This is of concern since accumulating research shows that early life FLX exposure results in long-term changes in gene expression [7, 8] and protein phosphorylation [9, 10] while altering responses to stress [10], memory [11] and/or reward-related behavior [12, 13]. In a recent study, we demonstrated that juvenile exposure to FLX, in female C57BL/6 mice, results in long-term decreases in the phosphorylation of extracellular signal-regulated kinase (ERK)-1/2 signaling markers within the prefrontal cortex (PFC) [14]. Likely mediating the anxiety-related response observed 21 days after juvenile FLX-exposure, since re-administration to FLX in adulthood restored both the anxiogenic-like phenotype as well as the decreases of ERK signaling within the PFC, but not the hippocampus. Interestingly, adult male rodents with a history of juvenile FLX exposure also display this anxiety-relevant phenotype [15]. Yet, whether similar long-term FLX-induced molecular alterations of ERK1/2-related signaling markers are evident within the male PFC has not been evaluated. For this reason, here, we examined for potential enduring FLX-induced changes in protein expression of ERK1/2, its downstream effector ribosomal S6 kinase (RSK), as well as mammalian target of rapamycin (mTOR) within this brain region—given the role these molecules play in modulating depression-related and antidepressant-like outcomes in adult organisms without juvenile antidepressant history [16–18].

## EXPERIMENTAL PROCEDURES

### Animals

**Figure 1:**
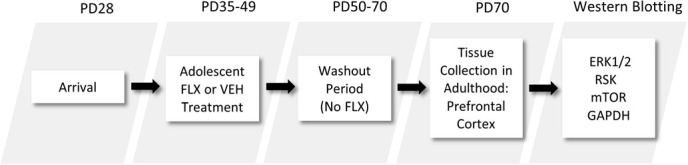
Timeline of adolescent fluoxetine (FLX) exposure and experimental procedures. Male Sprague-Dawley rats arrived to the animal facility at postnatal day (PD)-28. After 7 days, they were injected with FLX (20 mg/kg/day) or vehicle (VEH) for 15 days (PD35–49). Twenty-one days (long-term) after the last FLX injection (washout period), rats were euthanized for PFC tissue extraction and subsequent Western immunoblot analyses (PD70).

Experiments were conducted in compliance with the *Guide for the Care and Use of Laboratory Animals* [19], and with approval of the Institutional Animal Care and Use Committee. Postnatal day (PD) 28 male Sprague-Dawley rats were purchased from Charles River Laboratories (Hollister, CA, USA). Rats were housed in standard polypropylene cages containing wood shavings and placed on a 12:12 h light/dark cycle (lights on at 7 am) under unrestricted access to food and water. Rats were allowed 1 week of acclimation before the start of experiments (see Fig. 1).

### Antidepressant exposure and experimental design

FLX was purchased from Sigma-Aldrich (St. Louis, MI, USA), dissolved in sterile distilled water (vehicle; VEH), which served as control, and was administered in a volume of 2 mL/kg via intraperitoneal injections. Adolescent rats were administered FLX (20 mg/kg/day) or VEH for 15 days. The age and antidepressant regimen (PD35–49) was selected because it roughly approximates mid-adolescence in humans [20]. The FLX dose yields significant effects on behavior and gene expression relevant to antidepressant-related responses in preclinical models of stress [21, 22]. Twenty-one days (long-term) after the last FLX injection (PD70), rats were rapidly decapitated and PFC tissue was dissected on dry ice and stored at −80°C until assayed (see Fig. 1). Across the literature, it is well documented that rodents over 60 days of age are sexually mature and display decreases in play behavior [23–25]; therefore, we operationally define PD70 (i.e. age of tissue extraction) as adulthood in this investigation [26]. Additionally, the age of tissue extraction (PD70) matches prior work evaluating the long-term effects of antidepressant exposure during adolescence (PD35–49) when assessing molecular [9, 14] and behavioral outcomes across numerous domains, including responses to rewards [12, 27], stress [10, 15] and learning/memory mechanisms [11, 28].

### Western immunoblots

PFC tissue was sonicated in a standard lysis buffer and then centrifuged (14 000 rpm for 15 min). Samples (20 μg) were treated with β-mercaptoethanol and subsequently electrophoresed on precast 4–20% gradient gels (Bio-Rad), as previously published [14, 29]. Proteins were transferred to a polyvinylidene fluoride membrane, washed in 1X Tris-buffered saline with 0.1% Tween 20 (TBST) and blocked in milk dissolved in TBST (5% w/v) for 1 h at 25°C. Blots were probed overnight at 4°C with antibodies against the phosphorylated (p) forms of p-ERK1/2, p-RSK, p-mTOR and total (t)-glyceraldehyde-3-phosphate dehydrogenase (GAPDH), then stripped with Restore (Pierce Biotechnology) and re-probed with antibodies against t-ERK1/2 and t-mTOR. All antibodies were used according to the manufacturer’s instructions in 5% milk dissolved in TBST (antibodies listed in Supplemental Table 1). After additional washes, the membranes were incubated with peroxidase-labeled goat anti-rabbit IgG or horse anti-mouse IgG (1:40000; Vector Laboratories, Newark, CA, USA). Bands were visualized with SuperSignal West Dura substrate (Pierce Biotechnology), quantified using NIH ImageJ and normalized to GAPDH.

### Statistical analysis

Assignment of adolescent rats (PD35) to the different experimental conditions (FLX or VEH) was random. Two group comparisons were conducted using one-tail Student’s *t*-tests. Data are expressed as the mean ± standard error of the mean (SEM). Statistical significance was defined as *P* < 0.05.

## RESULTS

**Figure 2:**
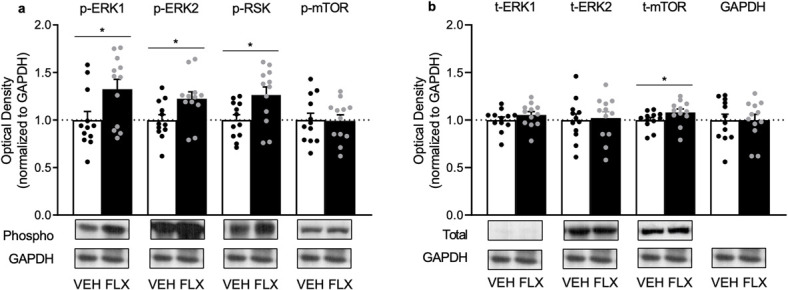
Enduring effects of fluoxetine (FLX) exposure during adolescence [postnatal day (PD) 35–49] on ERK1, ERK2, RSK and mTOR proteins in the prefrontal cortex of adult (PD70) male Sprague-Dawley rats. (**a**) Adolescent FLX history increased phosphorylated (p)-ERK1, p-ERK2 and p-RSK, without altering p-mTOR in adulthood (PD70). (**b**) Adolescent FLX history increased total (t) protein levels of mTOR, but not t-ERK1 or t-ERK2, 21 days after antidepressant pretreatment. Western blots of prefrontal cortex samples, with GAPDH as loading control, were performed under identical protocols. Representative images were cropped from different blots (Supplemental Fig. S1). ^*^P < 0.05. Data are presented as mean + SEM.

The effects of adolescent FLX exposure (PD35–49) on protein phosphorylation of ERK1/2, RSK and mTOR, within the PFC of adult male rats (PD70), are shown in Fig. 2a. When compared to VEH pretreated controls (*n* = 12), FLX pre-exposed rats (*n* = 12) displayed higher phosphorylation levels of ERK1 (t_22_ = 2.37, *P* = 0.0135), ERK2 (t_22_ = 2.43, *P* = 0.0117) and RSK (t_22_ = 2.57, *P* = 0.0086), but not mTOR (t_22_ = 0.09, *P* > 0.05). Figure 2b demonstrates how juvenile FLX history increases total protein levels of mTOR (t_22_ = 2.37, *P* = 0.0446), but not ERK1 or ERK2 (*P* > 0.05, respectively). No differences in GAPDH were evident between the groups (*P* > 0.05).

**Figure 3:**
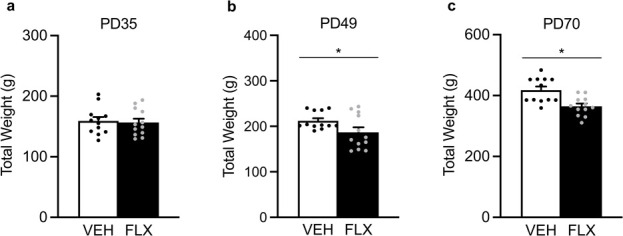
Acute and long-term effects of adolescent fluoxetine (FLX) exposure [postnatal day (PD) 35–49] on body weight-gain (g) in male Sprague-Dawley rats. (**a**) No differences in body weight were evident at the start of FLX exposure on PD35. (**b**) On the last day of antidepressant exposure (PD49), FLX-treated adolescent rats displayed lower body weight when compared to vehicle (VEH)-treated controls. (**c**) Twenty-one days after antidepressant exposure (PD70), adult rats pre-exposed to FLX during adolescence (PD35–49) continued to display lower body weight when compared to respective VEH controls. ^*^P < 0.05. Data are presented as mean + SEM.

Figure 3 shows the acute (PD49) and long-term (PD70) effects of juvenile FLX exposure on body weight (g) in male rats. No differences in body weight were noted at the start of FLX exposure when animals were PD35 (*P* > 0.05; Fig. 3a). However, on the last day of FLX exposure (PD49), FLX-exposed adolescent male rats displayed decreases in body weight (t_22_ = 2.11, *P* = 0.0461) when compared to VEH-controls (Fig. 3b). This FLX-induced decrease in body weight remained 21-days after antidepressant exposure, when animals reached adulthood (PD70; t_22_ = 3.58, *P* = 0.0008; Fig. 3c).

## DISCUSSION

Accumulating literature indicates that early life exposure to psychotropic medications is common [5, 30, 31] despite evidence that such pharmacological insult alters neurobehavior in adulthood [4, 5, 8, 27]. For example, juvenile FLX history alters mood-related behavioral outcomes in adult rodents, with FLX re-exposure normalizing these undesired phenotypes [14, 15] including a FLX-induced decrease in body weight [32]. Of note, in female mice, the enduring FLX-induced anxiety-like effect reported is accompanied by decreases of ERK-related signaling within the PFC, with such behavioral changes reversed by the re-introduction of FLX in adulthood (PD70+). This indicates that the PFC, like other brain regions [3, 8, 9], is vulnerable to long-term antidepressant-mediated molecular changes in later life. However, whether similar enduring FLX-induced ERK-related changes occur within the PFC of male rodents has not been assessed. For this reason, the present investigation evaluated the influence of adolescent FLX pre-exposure (PD35–49) on ERK1/2, RSK and mTOR protein expression within the adult PFC of male rats. We found that FLX pre-exposure increases the phosphorylation of ERK1/2 and RSK, as well as total protein levels of mTOR in this brain region at PD70 (Fig. 2). Thus, highlighting that sex is a critical factor for juvenile FLX-induced neurobiological changes within the adult PFC, given that decreases in ERK-related signaling markers are reported in female mice [14]. Interestingly, in both male rats and female mice, similar anxiety-relevant behavioral outcomes are observed 3 weeks after juvenile FLX exposure [14, 15]. This FLX-induced affect-related outcome, along with the present molecular findings, uncover both convergent (anxiety-related behavior) as well as divergent effects (oppositional PFC changes in ERK signaling) as a result of early life FLX exposure in a sex-specific manner. Yet, we must acknowledge that different rodent species were used between studies (male rats in the present investigation vs. female mice in [14]); therefore, it is possible that this long-term FLX-induced molecular signature could be species-specific, rather than sex-dependent.

Under normosensitive conditions, chronic stress decreases ERK in the PFC of adult male rats, with FLX administration restoring it back to normal levels [33]. For this reason, increases in PFC ERK-related molecules would be expected to result in resilient-like responses in adult animals with history of FLX pre-exposure. Indeed, this is the case when FLX-pretreated adult rodents are exposed to inescapable swim stress [15, 32] or social defeat episodes [10], emphasizing the functional role of PFC ERK1/2-RSK-mTOR signaling in regulating despair-related behavior [34]. Importantly, here, we extend increases of ERK1/2-RSK phosphorylation and mTOR protein levels, as a long-term PFC neurobiological FLX-induced effect, given that this molecular signature was evident 21 days post-treatment (Fig. 2). Yet, the literature suggests that adult rodents with a history of FLX exposure display a complex behavioral profile, wherein rodents show impairments in spatial memory [35] along with resilience to behavioral despair measures [32]. Paradoxically, these FLX-pretreated rodents spend less time in the open arms of the elevated plus maze—indicative of an anxiogenic-relevant outcome in adulthood [15]. While acute elevations of ERK-signaling in naïve adult male rats results in an anxiolytic-related behavioral outcome [36, 37], this oppositional effect may be mediated via long-term alterations of ERK-related markers within brain regions implicated in fear-learning mechanisms, since FLX was administered systemically in these studies. Indeed, ERK within the amygdala is negatively correlated with anxiety-related behavior [38] and, thus, it is likely that long-term ERK-signaling alterations within this brain region may be responsible for the anxiety-related behavioral phenotype reported in adult animals with a history of FLX exposure [3, 14, 15].

Since the PFC plays a critical role in various forms of learning and food seeking-related behavior [39], future work will be necessary to evaluate whether early life FLX results in long-term alterations in meal number/size consumption and/or changes in metabolism—particularly, because adolescent antidepressant pre-exposure decreases body weight in adulthood (Fig. 3c, 28, 32). Additionally, future investigations will be needed to determine if the increases in PFC ERK-related markers at PD70, as a function of adolescent FLX exposure (PD35–49), are sustained in later stages of adulthood. Lastly, whether upstream and/or downstream ERK-related molecular markers are altered differentially as a function of sex needs to be thoroughly evaluated, because it is well established that this signaling cascade mediates antidepressant-related outcomes via the regulation of both transcription and growth factors [i.e. ERK-cyclic AMP-response-element binding protein (CREB)-brain derived neurotropic factor (BDNF)] in a brain region-specific manner [33, 40–44].

Overall, we report that juvenile exposure to the SSRI FLX results in enhanced ERK-RSK-mTOR signaling activation within the PFC, along with persistent decreases in body weight, in adult male rats. The molecular and physiological findings of this investigation provide translational implications to uncover potential unknown long-term side effects that may result from juvenile SSRI treatment [6, 45].

## ACKNOWLEDGEMENTS

The authors would like to thank Paulina Vargas, Peter Fogel and Joselynn Reyes for technical assistance and maintenance of the *Iñiguez Behavioral Neuroscience Rodent Colony* at The University of Texas at El Paso (UTEP). Student co-authors acknowledge support to attain basic research experience/training per their respective program objectives. Specifically, M.R. acknowledges support from the Doctoral Excellence Fellowship (The Graduate School, UTEP). I.G.-C. acknowledges funding from the Louis Stokes Alliance for Minority Participation Bridge to Doctorate support program at UTEP.

## STUDY FUNDING

This work was supported by grants from the National Institute of General Medical Sciences from the National Institutes of Health (NIH-NIGMS: SC2GM109811 and 1SC3GM130467).

## CONFLICT OF INTEREST

The authors declare no financial or non-financial conflicts of interest. The funding agency was not involved in the experimental design, data collection process, interpretation of results, nor writing of the manuscript for publication.

## AUTHORS’ CONTRIBUTIONS

A.T. assisted with immunoblot experiments, analyzed data, interpreted results and co-wrote the manuscript. M.R., I.G.-C. and O.L. administered injections and assisted with the daily care of experimental animals. S.D.I. conceptualized and directed the project, assisted with immunoblot experiments, analyzed data, interpreted results and wrote the manuscript. All authors reviewed, edited and approved the manuscript.

## DATA AVAILABILITY STATEMENT

The data underlying this article will be shared on reasonable request to the corresponding author.

## Supplementary Material

Web_Material_kvac015
