# Supplementary material for: Adolescent fluoxetine exposure increases ERK-related signaling within the prefrontal cortex of adult male Sprague-Dawley rats
Source: Oxf Open Neurosci. 2022 Oct 20;1:kvac015. doi: 10.1093/oons/kvac015 (PMC9918101; doi:10.1093/oons/kvac015)
Supplement: Web_Material_kvac015 [file Web_Material_kvac015.zip › Supp.OxfordOpenNeuro.docx]

SUPPLEMENTAL MATERIAL

**Adolescent fluoxetine exposure increases ERK-related signaling within the prefrontal cortex of adult male Sprague-Dawley rats**

Anapaula Themann, Minerva Rodriguez, Israel Garcia-Carachure, Omar Lira, and *Sergio D. Iñiguez

Department of Psychology, The University of Texas at El Paso, El Paso, TX

*Corresponding Author: Sergio D. Iñiguez, Ph.D. (sdiniguez@utep.edu), Department of Psychology, The University of Texas at El Paso, 500 West University Avenue, El Paso, TX, 79968. Phone: 915-747-5769, Fax: 915-747-6553.

| **Supplementary Table 1**. Western Blot Primary Antibodies | | | |
| --- | --- | --- | --- |
| Antibody | Source | Company | Product # |
| p-ERK 1/2 | Rabbit | Cell Signaling (Danvers, MA) | 9101 |
| t-ERK 1/2 | Mouse | Cell Signaling (Danvers, MA) | 4696 |
| p-mTOR | Rabbit | Cell Signaling (Danvers, MA) | 2971 |
| t-mTOR | Mouse | Cell Signaling (Danvers, MA) | 4517 |
| p-p90RSK | Rabbit | Cell Signaling (Danvers, MA) | 9341 |
| GAPDH | Rabbit | Cell Signaling (Danvers, MA) | 2118 |

**
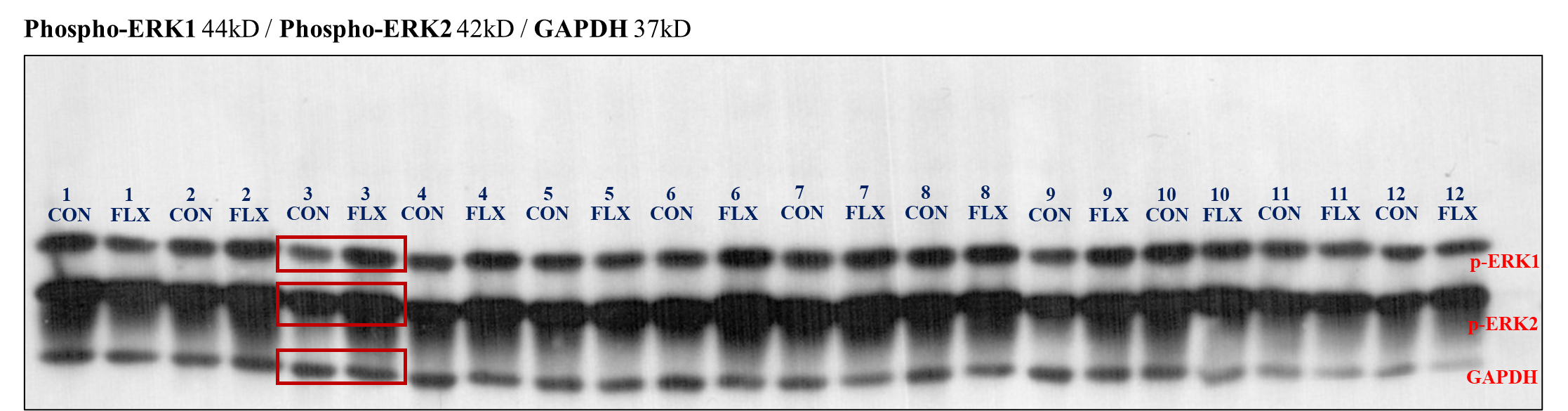
**

**
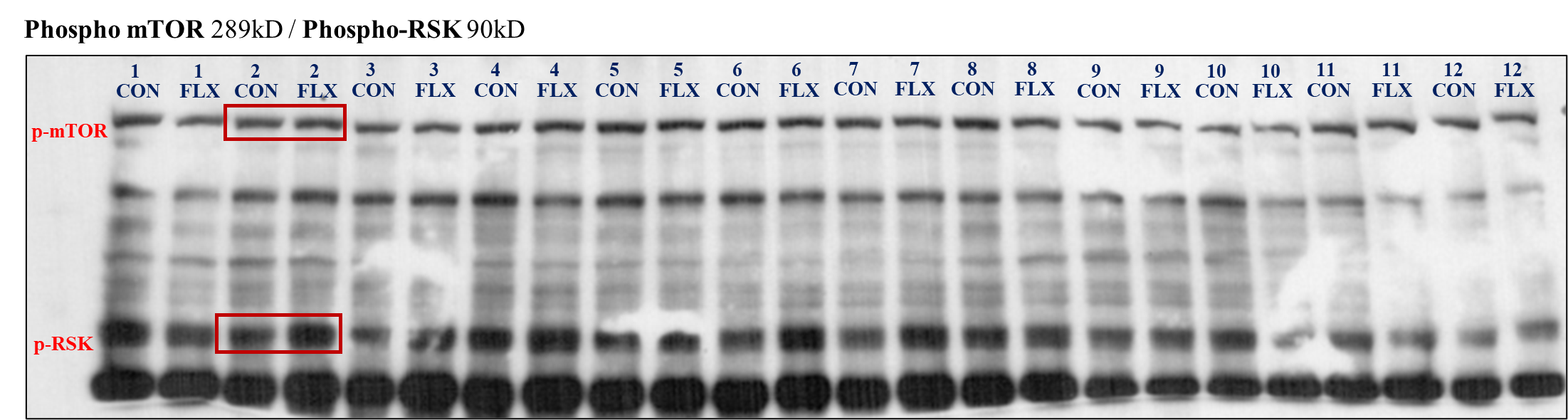
**

**
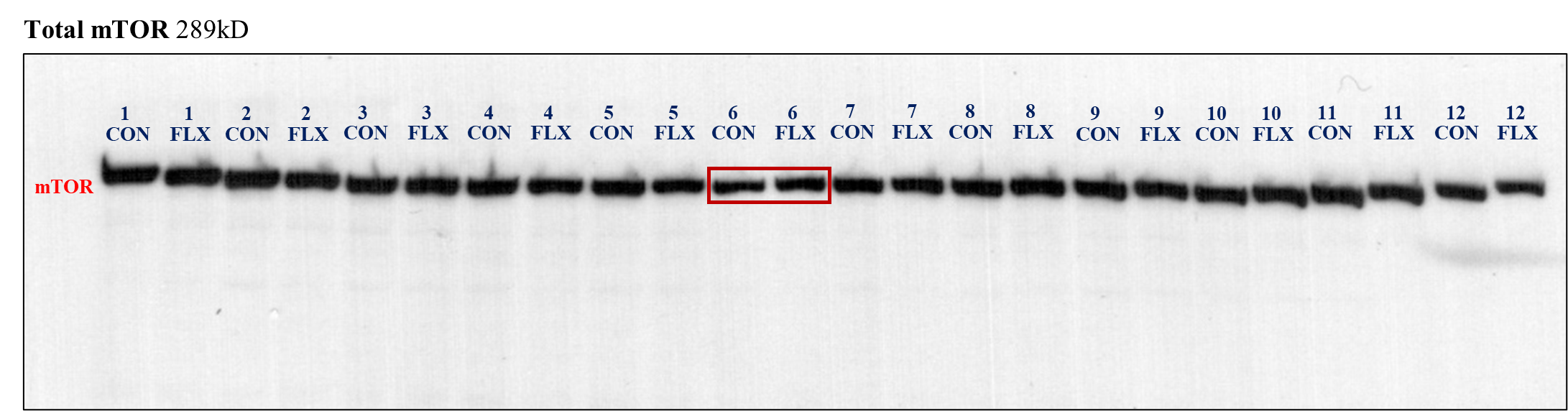
**


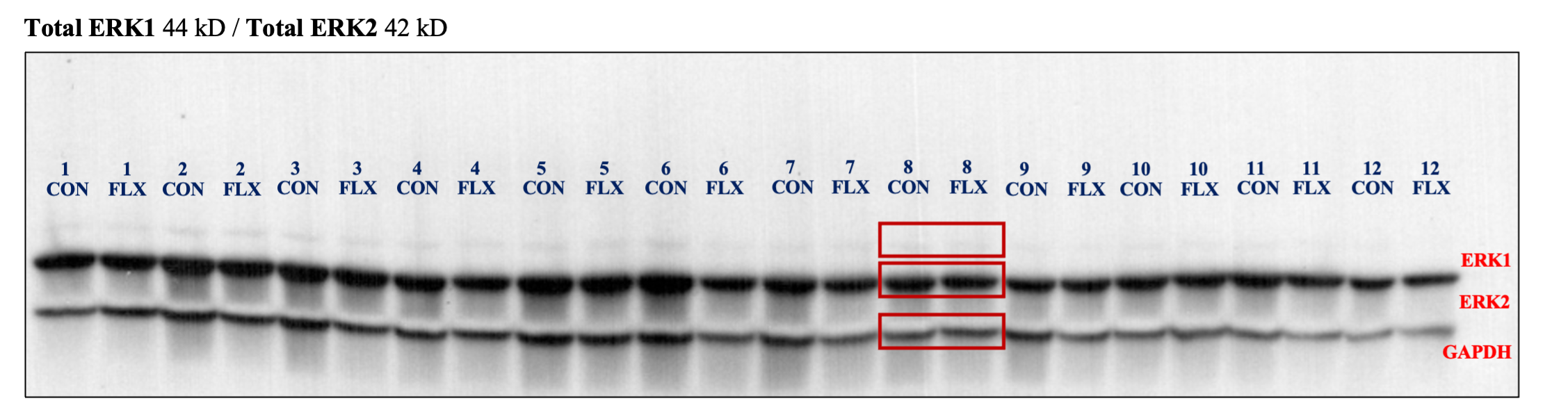


**Supplementary Figure S1.** Full blots of phospho (p)-ERK1/2, p-RSK, p-mTOR, or total (t)-ERK1/2, t-mTOR, and GAPDH prefrontal cortex proteins of postnatal day (PD70) male rats exposed to fluoxetine (FLX) or water-vehicle (CON) during adolescence (PD35-49). Selected wells (in red square) are featured as representative bands in Figure 2 of the manuscript.
