# Supplementary material for: Adolescent fluoxetine exposure increases ERK-related signaling within the prefrontal cortex of adult male Sprague-Dawley rats
Source: Oxf Open Neurosci. 2022 Oct 20;1:kvac015. doi: 10.1093/oons/kvac015 (PMC9918101; doi:10.1093/oons/kvac015)
Supplement: Web_Material_kvac015 [file Web_Material_kvac015.zip › Suppl. file.docx]

OXFNSC-2022-012.R1

Adolescent fluoxetine exposure increases ERK-related signaling within the prefrontal cortex of adult male Sprague-Dawley rats

Rapid communication

Reviewers’ comments

**Reviewer 1:**

Overview: the authors detail a rapid communication study that examined the effects of 2 weeks of treatment with the SSRI antidepressant fluoxetine on both longer-term weight gain and also protein markers for ERK1/2, RSK, and mTOR. The researchers reported that treatment with fluoxetine decreased body weight gain compared to control animals and increased measured protein levels when assessed 3 weeks after drug treatment ceased. The authors suggest these data may have implications for prefrontal function in developing adolescents and adults. I think the manuscript describes timely experiments with adequate analyses and interpretations. However, there are some areas of the manuscript that would benefit from additional information and clarification as detailed below.

1. This is a potentially important study. One significance of the current data may be that these results differ from those results have been reported in female animals. Thus, there may be a sex effect – although the present study was not designed to answer that question with direct comparison.

2. Age as a variable is an important factor to study but greater care needs to be done in justifying ages and their categorization. I think many would contend day 70 is not adulthood in rats. Some strains will show early puberty before day 30 and be fully fertile by day 60, so is that adulthood? I don’t think fertility status is a good metric – not that the current authors are making that argument -- but I am unclear what argument or metric they are using. They do make an argument for dosing at day 35-49, but not for decap and subsequent molecular methods. I think it is more common to see day 90 considered adulthood in many rat strains, but even then, I think you might find investigators arguing that day 100-120 is a better choice for the label of ‘adulthood.’ Perhaps a better strategy would be to choose timepoints that are when behavioral changes stop changing and are not close to the periods where phenotype changes are found. Unless of course, there are relevant behavioral timepoints that the authors could use to bolster their argument. I would be more comfortable if the authors modified their descriptors to label this day 70 stage as “late adolescence” instead of calling it “adulthood.”

3. Related question – is the 3 week time point after drug administration ended solely related to cumulative dosing washout of fluoxetine? Would we expect to see similar results at 4 weeks? 10 weeks? Or would there be recovery of function? The implications of these trajectories are very different (sensitive period vs chronic alteration) and warrant some consideration and discussion.

4. The authors should include exact p-values when reporting statistical analyses, when possible.

5. Probably a minor point, but were the drug- and veh-treated animals at equal weight to begin with at day 35? It might be helpful to include those data and analyses for the readers.

6. Also, following up on the points above, I don’t know if describing the continued observed difference in weight at 3 weeks later as “long term” is the best phrasing (Fig 3 + pg 7, line 13)

7. The authors conclude with an overarching statement questioning the safety of juvenile SSRI treatment. I believe there are documented differences in effects of SSRIs and antidepressants on these molecular activation pathways, thus it would be better to be a bit more circumspect with phrasing.

Minor points of consideration

1. It is helpful to define all abbreviations the first time used

2. The most recent edition of the NRC’s Guide for the Care and Use of Animals is 2011

**Reviewer 2:**

In this manuscript, the authors investigate the long-term effects of juvenile fluoxetine (FLX) exposure on the phosphorylation and expression of ERK1/2, RSK and mTOR in the PFC of male rats. The aim of their study is clear and addresses a significant question, i.e., whether FLX administration in juveniles is safe. The results are straightforward and convincing and open up future research directions.

- The authors state that 20mg/kg of FLX is a dose that yields significant effects on rodent behaviour. Could the authors also state how this dose compares to the dose used in humans?

- In their previous publication (Flores-Ramirez et al., 2021), the authors reported that juvenile exposure to FLX lead to a decrease in ERK-related signaling in the PFC of female mice. In the present study male rats exposed to juvenile FLX showed an increase in ERK-related signaling. Since these two studies have been performed on different species (mouse and rat), it is difficult to interpret whether the opposite effects on ERK signaling are due to sex or species differences. Could the authors elaborate why they chose to perform these experiments on male rats rather than mice like in their previous study?

- Could the authors briefly address the mechanism by which the ERK-related signaling pathway mediates anti-depressive effects?

- Whilst this is out of the scope of this “Rapid Communication” manuscript, it would be of interest to test whether the increase in ERK phosphorylation in juvenile FLX treated male rats is also accompanied by a decrease in anxiogenic behaviour.

Minor text editing comments

- Section ‘Introduction’, Row 33: a space between the ‘-‘ and ‘predominantly’

- Section ‘Animals’, Row 22: ‘acclimation’ instead of ‘acclamation’

- Section ‘Western Immunoblots’, Row 10: ‘described.’

**Decision letter**

08-Sep-2022

Dear Dr. Iñiguez,

Manuscript ID OXFNSC-2022-012 entitled "Adolescent fluoxetine exposure increases ERK-related signaling within the prefrontal cortex of adult male Sprague-Dawley rats" which you submitted to the Oxford Open Neuroscience, has been reviewed. The comments of the reviewer(s) are included at the bottom of this letter.

Please note that this journal operates with transparent peer review. This means that if your submission is accepted for publication, the full peer review history of your article will publish online alongside your article. This includes reviewer comments, editor decision letters, and your author responses.

The reviewer(s) have recommended publication, but also suggest some major revisions to your manuscript. Therefore, I invite you to respond to the reviewer(s)' comments and revise your manuscript.

To revise your manuscript, log into https://mc.manuscriptcentral.com/oxfnsc and enter your Author Centre, where you will find your manuscript title listed under "Manuscripts with Decisions." Under "Actions," click on "Create a Revision." Your manuscript number has been appended to denote a revision.

You may also click the below link to start the revision process (or continue the process if you have already started your revision) for your manuscript. If you use the below link you will not be required to login to ScholarOne Manuscripts.

*** PLEASE NOTE: This is a two-step process. After clicking on the link, you will be directed to a webpage to confirm. ***

https://mc.manuscriptcentral.com/oxfnsc?URL_MASK=c20dad0660bb4da3b34b1f2790eb0d62

You will be unable to make your revisions on the originally submitted version of the manuscript. Instead, revise your manuscript using a word processing program and save it on your computer. Please also highlight the changes to your manuscript within the document by using the track changes mode in MS Word or by using bold or colored text.

Once the revised manuscript is prepared, you can upload it and submit it through your Author Centre.

When submitting your revised manuscript, you will be able to respond to the comments made by the reviewer(s) in the space provided. You can use this space to document any changes you make to the original manuscript. In order to expedite the processing of the revised manuscript, please be as specific as possible in your response to the reviewer(s).

IMPORTANT: Your original files are available to you when you upload your revised manuscript. Please delete any redundant files before completing the submission.

Because we are trying to facilitate timely publication of manuscripts submitted to the Oxford Open Neuroscience, your revised manuscript should be uploaded as soon as possible. If it is not possible for you to submit your revision in a reasonable amount of time, we may have to consider your paper as a new submission.

Once again, thank you for submitting your manuscript to the Oxford Open Neuroscience and I look forward to receiving your revision.

Sincerely,

Dr. Hongyan Wang

Senior Editor, Oxford Open Neuroscience

hongyan.wang@duke-nus.edu.sg

Associate Editor

Comments to the Author:

Dear authors,

By now we have received two reviews of your manuscript and I am happy to inform you that both reviewers are mostly positive about your study (see below). They however raised a few issues that I am asking you to address in full. We are looking forward to receive your revision within six weeks but please let us know if this time frame may not be suitable so that we can extend it in case necessary.

Sincerely,

Simon HIPPENMEYER

Associate Editor, Oxford Open Neuroscience

simon.hippenmeyer@ist.ac.at

**Authors’ response**

We thank the reviewers for their positive and constructive feedback on our original submission. We have addressed each of their points individually below, and the corresponding changes to the manuscript are shown in blue.

Reviewer #1

1.1 “This is a potentially important study. One significance of the current data may be that these results differ from those results have been reported in female animals. Thus, there may be a sex effect – although the present study was not designed to answer that question with direct comparison.”

We thank the reviewer for taking the time to carefully evaluate our manuscript, and for highlighting the importance of our experimental work. In a recent publication, we reported that adolescent FLX exposure results in long-term decreases of molecular markers from the extracellular signal-regulated protein kinase (ERK) cascade within the prefrontal cortex (PFC) of female mice (Flores-Ramirez et al., Sci Reports, 2021). However, whether similar enduring ERK-related changes are evident in male rodents had not been evaluated. For this reason, we tested if adolescent FLX exposure altered PFC ERK-RSK-mTOR signaling three-weeks post antidepressant exposure in male Sprague-Dawley rats. We found that juvenile FLX exposure resulted in long-term increases of all three molecular markers, thus, uncovering a differential molecular signature in males as a function of FLX. As the reviewer highlights, our experimental design does not allow direct sex comparisons. To avoid confusion, we indicate in the Introduction section of the paper that our approach stems from what has been previously reported in females (see end of Page 2 and beginning of Page 3). Briefly, we clarify that our experimental approach is hypothesis driven, while not incorporating “sex” as an independent variable.

1.2 “Age as a variable is an important factor to study but greater care needs to be done in justifying ages and their categorization. I think many would contend day 70 is not adulthood in rats. Some strains will show early puberty before day 30 and be fully fertile by day 60, so is that adulthood? I don’t think fertility status is a good metric – not that the current authors are making that argument -- but I am unclear what argument or metric they are using. They do make an argument for dosing at day 35-49, but not for decap and subsequent molecular methods. I think it is more common to see day 90 considered adulthood in many rat strains, but even then, I think you might find investigators arguing that day 100-120 is a better choice for the label of ‘adulthood.’ Perhaps a better strategy would be to choose timepoints that are when behavioral changes stop changing and are not close to the periods where phenotype changes are found. Unless of course, there are relevant behavioral timepoints that the authors could use to bolster their argument. I would be more comfortable if the authors modified their descriptors to label this day 70 stage as “late adolescence” instead of calling it “adulthood.”

As stated by the reviewer, it is well established across the literature that rodents older than 60 days of age are conventionally classified as adults (for examples, see: Laviola et al., Neurosci Biobehav Rev,27(1-2):19-31, 2003; Eiland & Romeo, Neuroscience, 249: 162-171, 2013; McCormick et al., Brain Cogn, 72:73-85, 2010; Tirelli et al, Neurosci Biobehav Rev, 27(1-2):163-178, 2003; Andersen SL, Neurosci Biobehav Rev, 27: 3-18, 2003). Both Sprague-Dawley rats and C57BL/6 mice are sexually mature post 60 days of age, and display adult-like behavior (i.e., decreased play and/or risk-taking behavior). Therefore, we operationally define postnatal day 70 animals as adults in our study (see Page 4, Lines 6-12; see also Abstract on Page 1, Line 6). We believe this is a reasonable classification, particularly, because we are adopting the same age of antidepressant exposure, as well as the behavioral/molecular endpoint (postnatal day 70) as prior published work (Flores-Ramirez et al., Sci Rep 11(1):7758, 2021; Sierra-Fonseca et al. J Alzheimers Dis, 83(4):1691-1702, 2021); thus, directly expanding on this understudied topic. However, we do agree with the reviewer that additional age groups (greater than PD70) should also be studied (since adulthood is a broad non-specific term). For this reason, we now indicate that future work should incorporate such experimental approach in the Discussion section of the manuscript (see 2nd paragraph on Page 8, Lines 12-14).

1.3 “Related question – is the 3 week time point after drug administration ended solely related to cumulative dosing washout of fluoxetine? Would we expect to see similar results at 4 weeks? 10 weeks? Or would there be recovery of function? The implications of these trajectories are very different (sensitive period vs chronic alteration) and warrant some consideration and discussion.”

The half-life of FLX is 4-6 days, and 4-16 days for its metabolite norfluoxetine - given its high affinity for the serotonin transporter. Because we wait 21 days post FLX exposure, we argue that the molecular changes observed are long-lasting (a term that is operationally defined across the body of the manuscript and Figure captions). Of course, we value the suggestion of expanding this line of work to longer periods of time (i.e., later adulthood). Thus, we now indicate in the Discussion section of the paper that such experimental approach should be pursued in the future (Page 8, Line 12-14).

1.4 “The authors should include exact p-values when reporting statistical analyses, when possible.”

As recommended, we have included the exact p-value whenever is lower than 0.05 (see Results section of the paper on Pages 5-6).

1.5 “Probably a minor point, but were the drug- and veh-treated animals at equal weight to begin with at day 35? It might be helpful to include those data and analyses for the readers.”

We apologize for occluding this important piece of information in our original submission. Importantly, no differences in body weight were evident at the start of FLX treatment (postnatal day 35). We now include this data in the updated Figure 3.

1.6 “Also, following up on the points above, I don’t know if describing the continued observed difference in weight at 3 weeks later as “long term” is the best phrasing (Fig 3 + pg 7, line 13).”

We operationally define long-term as a 3-week washout period in the Methods section of the paper (Page 4, Line 4) as well as in the caption of Figure 1 when describing our experimental approach. To avoid confusion, we also repeat this across numerous sections of the paper.

1.7 “The authors conclude with an overarching statement questioning the safety of juvenile SSRI treatment. I believe there are documented differences in effects of SSRIs and antidepressants on these molecular activation pathways, thus it would be better to be a bit more circumspect with phrasing.”

We value the comment from the reviewer and agree that we should be more careful when interpreting the results of our experiment. We have modified the last sentence of the Discussion section to indicate that enduring molecular changes should be expected as a function of FLX history, rather than questioning the safety of SSRI used (see last sentence of the Abstract, as well as the last sentence of the manuscript’s Discussion section on Page 9).

1.8 “Minor points of consideration

a) It is helpful to define all abbreviations the first time used

b) The most recent edition of the NRC’s Guide for the Care and Use of Animals is 2011.”

1.8a We thank the reviewer for catching the inconsistency. We have now ensured that all acronyms are defined when initially introduced in both Abstract and body of the paper. If we accidentally missed any, we welcome specific examples for us to modify.

1.8b As recommended, we have updated the reference of the Guide for the Care and Use of Animals to the most recent version published in 2011 (see updated reference 19).

Reviewer #2

2.1 “The authors state that 20mg/kg of FLX is a dose that yields significant effects on rodent behaviour. Could the authors also state how this dose compares to the dose used in humans?”

We thank the reviewer for the insightful comments geared toward making this a better and more cohesive manuscript. Prior work in male rodents shows that 20 mg/kg results in similar FLX plasma levels as the doses commonly used in humans (20-80 mg; Dulawa et al., Neuropsychopharmacology, 29(7):1321-30, 2004). Importantly, this dose decreases both juvenile and adult rat immobility in the forced swim test, a traditional animal model for the study of antidepressant efficacy (Iñiguez et al, Biol Psychiatry, 67(11):1057-66, 2010). As such, it is likely that the enduring molecular changes reported in our work are not mediated by differential blood serum concentrations between humans and rodents (see Dulawa et al., Neuropsychopharmacology, 29(7):1321-30, 2004).

2.2 “In their previous publication (Flores-Ramirez et al., 2021), the authors reported that juvenile exposure to FLX lead to a decrease in ERK-related signaling in the PFC of female mice. In the present study male rats exposed to juvenile FLX showed an increase in ERK-related signaling. Since these two studies have been performed on different species (mouse and rat), it is difficult to interpret whether the opposite effects on ERK signaling are due to sex or species differences. Could the authors elaborate why they chose to perform these experiments on male rats rather than mice like in their previous study?”

We selected to use Sprague Dawley rats to directly expand on our prior work - wherein we evaluated the long-term effects of FLX across numerous mood-related behavioral phenotypes in male rats (Iñiguez et al, Biological Psychiatry, 67(11):1057-66, 2010). However, we do agree with the reviewer that it is possible that the sex-specific differences noted/discussed on ERK-related signaling, as a result of juvenile FLX exposure, could be species-specific and not sex-dependent. To avoid confusion, we now highlight this limitation in the Discussion of the manuscript (see Page 7, Lines 6-9).

2.3 “Could the authors briefly address the mechanism by which the ERK-related signaling pathway mediates anti-depressive effects?”

We thank the reviewer for bringing this to our attention. We now highlight that ERK regulates antidepressant-like responses via the activation of CREB-BDNF signaling. We reference studies that display this relationship within several brain regions, including the PFC and hippocampus (see Page 8, Lines 16-19), while indicating that future work is needed to evaluate upstream and downstream ERK-related molecules post FLX exposure as a function sex.

2.4 “Whilst this is out of the scope of this “Rapid Communication” manuscript, it would be of interest to test whether the increase in ERK phosphorylation in juvenile FLX treated male rats is also accompanied by a decrease in anxiogenic behaviour.”

The reviewer makes a keen observation. In normal adult male rats, increasing ERK signaling results in an anxiolytic-like behavioral phenotype, per the elevated plus mase. Yet, adult rats with a history of juvenile FLX exposure display an anxiogenic-like profile when evaluated on this same task. We now address this paradoxical effect in the Discussion section (see last sentence on Page 7, and first paragraph on Page 8).

2.5 “Minor text editing comments

a) Section ‘Introduction’, Row 33: a space between the ‘-‘ and ‘predominantly’

b) Section ‘Animals’, Row 22: ‘acclimation’ instead of ‘acclamation’

c) Section ‘Western Immunoblots’, Row 10: ‘described.”

2.5a Thank you for catching this. We have now ensured there is a single space between sentences across the manuscript.

2.5b Thank you for catching the typo. We have changed the word to acclimation.

2.5.c We have modified the sentence to indicate that we followed previously published work (rather than described).

**Decision letter**

08-Oct-2022

Dear Dr. Iñiguez,

It is a pleasure to accept your revised manuscript entitled "Adolescent fluoxetine exposure increases ERK-related signaling within the prefrontal cortex of adult male Sprague-Dawley rats" in its current form for publication in the Oxford Open Neuroscience. The comments of the reviewer(s) who reviewed your manuscript are included at the foot of this letter.

Please note that this journal operates with transparent peer review. This means that the full peer review history of your article will publish online alongside your article. This includes reviewer comments, editor decision letters, and your author responses.

Thank you for your fine contribution. On behalf of the Editors of the Oxford Open Neuroscience, we look forward to your continued contributions to the Journal.

Sincerely,

Dr. Hongyan Wang

Senior Editor, Oxford Open Neuroscience

hongyan.wang@duke-nus.edu.sg

Dr. Simon Hippenmeyer

Associate Editor, Oxford Open Neuroscience

simon.hippenmeyer@ist.ac.at

Reviewer: 2

Comments to the Author

The authors answered all my questions satisfactorily and made the corresponding changes in the manuscript.

Reviewer: 1

Comments to the Author

The authors have adequately dealt with all of my earlier comments. I have no further commentary. Thank you for continuing to investigate the many effects of fluoxetine.
